# Diffusion Tensor Based White Matter Tract Atlases for Pediatric Populations

**DOI:** 10.3389/fnins.2022.806268

**Published:** 2022-03-23

**Authors:** Sarah J. Short, Dae Kun Jang, Rachel J. Steiner, Rebecca L. Stephens, Jessica B. Girault, Martin Styner, John H. Gilmore

**Affiliations:** ^1^Department of Educational Psychology, University of Wisconsin-Madison, Madison, WI, United States; ^2^Center for Healthy Minds, University of Wisconsin-Madison, Madison, WI, United States; ^3^Department of Psychiatry, University of North Carolina at Chapel Hill, Chapel Hill, NC, United States; ^4^Carolina Institute for Developmental Disabilities, University of North Carolina at Chapel Hill, Chapel Hill, NC, United States; ^5^Department of Computer Science, University of North Carolina at Chapel Hill, Chapel Hill, NC, United States

**Keywords:** infant, pediatric, DTI, neuroimaging, computational atlas, white matter tracts, MRI

## Abstract

Diffusion Tensor Imaging (DTI) is a non-invasive neuroimaging method that has become the most widely employed MRI modality for investigations of white matter fiber pathways. DTI has proven especially valuable for improving our understanding of normative white matter maturation across the life span and has also been used to index clinical pathology and cognitive function. Despite its increasing popularity, especially in pediatric research, the majority of existing studies examining infant white matter maturation depend on regional or white matter skeleton-based approaches. These methods generally lack the sensitivity and spatial specificity of more advanced functional analysis options that provide information about microstructural properties of white matter along fiber bundles. DTI studies of early postnatal brain development show that profound microstructural and maturational changes take place during the first two years of life. The pattern and rate of these changes vary greatly throughout the brain during this time compared to the rest of the life span. For this reason, appropriate image processing of infant MR imaging requires the use of age-specific reference atlases. This article provides an overview of the pre-processing, atlas building, and the fiber tractography procedures used to generate two atlas resources, one for neonates and one for 1- to 2-year-old populations. *Via* the UNC-NAMIC DTI Fiber Analysis Framework, our pediatric atlases provide the computational templates necessary for the fully automatic analysis of infant DTI data. To the best of our knowledge, these atlases are the first comprehensive population diffusion fiber atlases in early pediatric ages that are publicly available.

## Introduction

Diffusion-Weighted Imaging (DWI) is a magnetic resonance imaging (MRI) technique that measures the diffusion of water molecules to characterize microstructural aspects of the brain. Diffusion rates of water molecules differ by brain tissue type, and thus, DWI allows researchers to examine the structural properties and connectivity of white matter fiber tracts. Along with the DWI technique, Diffusion Tensor Imaging (DTI) provides a means to analyze the diffusion tensor, which is the three-dimensional shape of the water diffusion, enabling the 3D visualization of white matter fiber pathways.

Researchers utilize DWI and DTI techniques to build a 3D atlas that can provide a standardized reference space for anatomical guidance. Such reference spaces are considered crucial for consistency, reliability, and reproducibility of neuroimaging studies. A DTI atlas can serve as a tool for identifying changes in white matter tracts and for assessing the neurodevelopment of both healthy and at-risk children using, for example, tract-oriented statistics. Moreover, the use of an atlas can also enhance the diagnostic utility of neuroimaging and can quantitatively identify aberrant developmental patterns related to clinical conditions (Oishi et al., 2013).

To date, however, most atlases have been developed for the adult human brain (McLaren et al., 2009; Adluru et al., 2012), and many previous neonate studies (Geng et al., 2012a; Loh et al., 2012) relied on atlases built for adults or older children. Considering that one of the advantages of DWI and DTI techniques is the opportunity to study neurodevelopmental patterns of white matter microstructure, it is questionable to use an atlas of the adult human brain as the reference for neonatal and pediatric studies. Even to the untrained eye of a layperson, the anatomical features of the brain differ greatly during early development, especially during the first few years of life when there are dramatic changes in the size, shape, and degree of gray matter, white matter, and cerebral spinal fluid (Wilke and Holland, 2003; Kuklisova-Murgasova et al., 2011). Early brain development is a dynamic and rapid process. From birth to 2 years of age, anatomical architecture becomes highly connected and organized, and the brain undergoes its most rapid growth of any point during the life span (Ouyang et al., 2019). Thus, a mismatch in age between atlas and research participants may result in poor registration accuracy and increased measurement error, potentially limiting the reproducibility, reliability, and translation of findings both within and beyond the data set.

The application of DTI analysis methods in infants also has the potential to enhance our understanding of the typical variation in brain development patterns and the structural correlation of rapidly developing cognitive capabilities. This potential is especially meaningful as it may enable the development of a model for monitoring white matter maturation as it relates to the observable behavioral and structural changes predictive of neurodevelopmental or psychiatric disorders.

Some research groups have endeavored to build age-specific brain atlases for studies of neonates to overcome the aforementioned issues, although they are few in number and most are not publicly available. One exception however comes from Oishi et al. (2019) who created two types of infant atlases that are publicly available: a single-subject atlas (Habas et al., 2010a,b; Kuklisova-Murgasova et al., 2011; Shi et al., 2011; Serag et al., 2012; Akiyama et al., 2013; Li et al., 2015; Makropoulos et al., 2016; Zhang et al., 2016) and a multi-atlas (Gousias et al., 2012; Blesa et al., 2016; Alexander et al., 2017; Otsuka et al., 2017). The purpose of these atlases is to provide reliable regional reference labels of brain anatomy using parcellation maps. The parcellation maps not only provide the means to research structural connectivity but also aid in the examination of the functional brain connectivity. To be more specific, they utilize white matter regional or skeleton-based analyses that contain multiple white matter structures within a seed region of interest (ROIs), which are identified using the anatomical information from the parcellation maps (Oishi et al., 2019). However, these regional analysis methods generally lack the sensitivity of a joint multivariate analysis of diffusion properties, as well as lack the spatial specificity of more advanced analysis along fiber bundles identifying not just an individual fiber bundle, but the specific anatomical location along it.

To address these limitations, one can employ an alternate means of structural analysis *Via* tractography of white matter fiber bundles to examine neonatal and pediatric DTI data, such as in the UNC-NAMIC DTI Fiber Analysis Framework (Verde et al., 2014). DTI tractography is a 3D modeling technique used to visualize microstructural properties of white matter fiber bundles. This non-invasive tool provides *in vivo* dissections of specific neural pathways that can be correlated with physical or mental function (Lawes et al., 2008). Furthermore, fiber tract-based analysis enables scientists to perform functional statistical analyses along the fiber bundle by extracting profiles of diffusion properties.

Despite the wealth of its applications, the fiber-tract based analysis approach is not widely applied in the study of neonatal DTI data. This is likely due to the challenges of large-scale DTI data acquisition in non-sedated newborns, coupled with the computational complexity of necessary analysis approaches, as well as the lack of available DTI fiber atlases. To assist with some of these issues, we present here two publicly available pediatric DTI atlases based on comprehensive diffusion imaging, extensive quality control, accurate atlas building, and time-intensive interactive tractography for white matter tract dissections. Below, we report an overview of the data pre-processing, atlas building, and tractography procedures used for the construction of two developmentally appropriate atlases specific to neonatal and pediatric populations.

## Materials and Methods

### Participants

The UNC Early Brain Development Study (EBDS) database is a unique and innovative longitudinal dataset that has followed children, enrolled prenatally, with imaging and cognitive/behavioral assessments throughout their postnatal brain development. From the EBDS database, we selected participant scans collected within a few weeks of birth, and at 1- and 2-years of age from two ongoing longitudinal studies: a study of normative development that also includes children at genetic risk for psychiatric disorders (Gilmore et al., 2010a); and a twin study of early brain development (Gilmore et al., 2010b). We obtained informed consent from the parents of all participants, and all protocols were approved by the Institutional Review Board at the University of North Carolina at Chapel Hill. Participants included in the two atlases were selected without consideration of gender, medical conditions, demographic information, and other forms of patient protected data (PHI).

The first atlas, the EBDS Neonatal DTI Atlas, was constructed from 144 participants, born between 30 and 42 weeks gestation (37.11 ± 2.6), with DWI scans acquired within the first few weeks of life, or between 38 and 48 weeks gestational age (41.88 ± 1.83). Participant demographic information for scans included in the neonatal atlas can be found in Table 1.

**TABLE 1:** Demographic characteristics for participants included in neonatal atlas.

	N	
**Child sex**		
Male	68	
Female	76	
**Race**		
White	98	
Black or African American	25	
Other	21	
**Twin status**		
Non-twin (singleton)	71	
Twin	73	
Monozygotic	27	
Dizygotic	46	

	**M (SD)**	**Range**

Gestational age at birth (weeks)	37.11 (2.66)	30.14 – 42.14
Birthweight (g)	2822.84 (682.57)	1060 – 4730
Gestational age at scan (PMA, weeks)	41.88 (1.83)	38.14 – 48
Age at scan (days since birth)	30.83 (16.12)	10 – 86
Maternal education (years)	14.98 (3.98)	3 – 24
Maternal age at birth (years)	29.27 (5.10)	16 – 42

The second atlas, the EBDS Pediatric DTI Atlas, was constructed from 341 scans acquired from 295 unique typically developing 1-year (*n* = 170) and 2-year-old participants (*n* = 171), born between 27 and 42 weeks gestation (37.20 ± 2.7), with DWI scans acquired at an average age of 13.03 (±0.95) months for the 1-year-olds and 25.05 (±1.07) months for the 2-year-olds. Participant demographic information for scans included in the pediatric atlas can be found in Table 2.

**TABLE 2:** Demographic characteristics for participants included in pediatric atlas.

	1-year scans (*n* = 170)		2-year scans (*n* = 171)	
	*N*		*N*	
**Child sex**				
Male	95		88	
Female	75		83	
**Race**				
White	97		100	
Black or African American	60		58	
Other	13		13	
**Twin status**				
Non-twin (singleton)	80		80	
Twin	90		91	
Monozygotic	34		44	
Dizygotic	56		46	

	**M (SD)**	**Range**	**Mean (SD)**	**Range**

Gestational age at birth (weeks)	37.12 (2.77)	27.43 – 41.29	36.95 (2.88)	27.43 – 42.14
Birthweight (g)	2795.34 (721.01)	790 – 4730	2763.78 (738.03)	790 – 4701
Gestational age at scan (PMA, weeks)	93.72 (0.52)	86.29 – 109.60	108.87 (4.67)	131.43 – 162.43
Age at scan (weeks since birth)	56.60 (0.59)	47.43 – 73.00	145.82 (3.84)	94.43 – 121.14
Maternal education (years)	14.05 (3.95)	0 – 25	13.69 (3.40)	0 – 23
Maternal age at birth (years)	29.70 (6.59)	17 – 45	29.81 (6.09)	16 – 45

All participants for the atlas building were selected based on the following criteria for inclusion: processed diffusion tensor data was free from artifacts, at a high level of SNR, and adequate reconstruction of major white matter fiber bundles established *Via* interactive tractography.

### Image Acquisition

All neonatal and pediatric MRI scans were acquired on 3T Siemens MR scanners (Siemens Medical Solutions, Erlangen, Germany), BRIC, Chapel Hill, NC, United States using one of two different scan sequences: a 6-direction or a 42-direction sequence on the Siemens Allegra scanner and the same 42-direction protocol on Siemens Tim Trio scanner.

All participants were scanned without sedation during natural sleep. Before scanning, neonate participants were fed and swaddled. Scans for 1- and 2-year-old participants were scheduled to take place just prior to nap/sleep time. Once asleep, participants were fitted with earplugs and earphones and placed in the MRI scanner with their heads in a vacuum-fixation device. We performed neonate scans with a neonatal nurse present, and a pulse oximeter to monitor heart rate and oxygen saturation. Scans for the older children were performed with a research team member present in the scanner room throughout the scan to monitor the child.

#### Six Direction Sequence (Allegra)

A single-shot, twice refocused echo-planar spin echo DTI sequence was used with the following variables: TR 5,200 ms, TE = 73 ms, slice thickness = 2 mm, in-plane resolution = 2 mm × 2 mm, and 45 slices. One image without diffusion gradients (b = 0, b0) and DWIs along 6 gradient directions, with a *b* value of 1,000 s/mm^2^, were acquired. The acquisition was repeated five times to improve signal-to-noise ratio.

#### Forty-two Direction Sequence (Allegra and Trio)

A single-shot, twice refocused echo-planar spin echo DTI sequence was used with the following variables: TR = 7680 ms, TE = 82 ms, slice thickness = 2 mm, in-plane resolution = 2mm × 2mm, a total of 49 DWI volumes were acquired, 7 without diffusion gradients (b = 0, b0) and 42 with b = 1000 s/mm^2^ in unique directional diffusion gradients.

### Methods Overview

**FIGURE 1:**
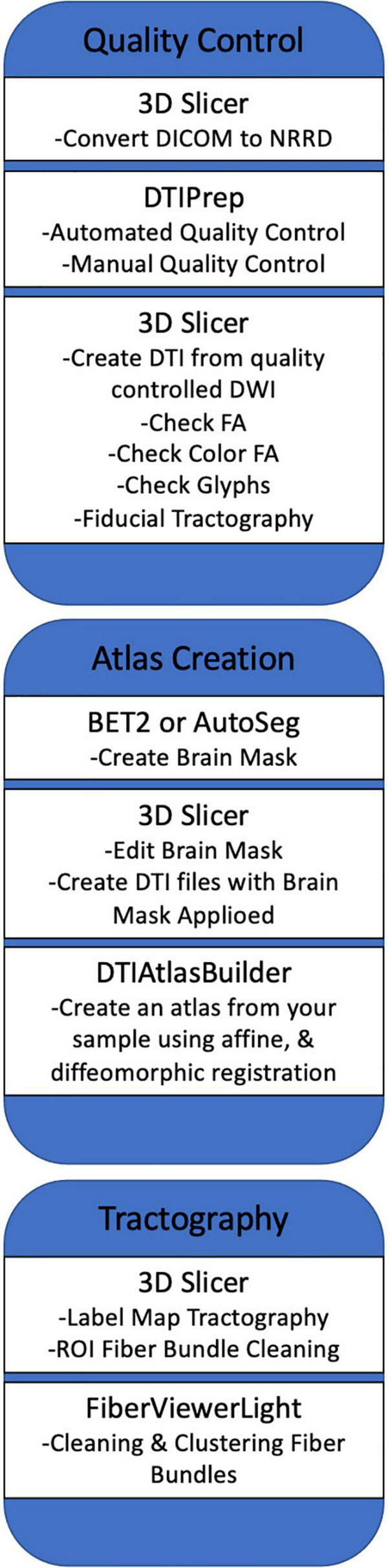
A summarized overview of the UNC Utah NAMIC framework. The figure outlines the framework from quality control steps to statistical analysis of white matter tracts.

Our framework for the fiber tract-based analysis of DTI data closely follows the UNC Utah NAMIC pipeline (Verde et al., 2014, 2013), with age-specific modifications to accommodate the unique characteristics of diffusion imaging the pediatric brain. This framework consists of the following components: (1) comprehensive quality control and pre-processing to remove bad MRI data, (2) brain masking, (3) accurate DTI atlas building, and (4) expert-guided, interactive tractography in the atlas for white matter tract dissection. These steps are discussed below in more detail. A summarized overview of the framework can be seen in Figure 1.

### Pre-processing and Quality Control

**FIGURE 2:**
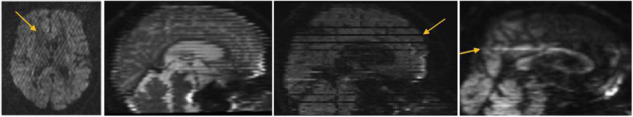
Commonly observed diffusion MRI artifacts, particularly in infant scans. Our diffusion MRI preprocessing with DTIPrep removes all these artifacts, indicated with arrows, from the scans prior to further processing.

Using DTIPrep (Liu et al., 2015; v.1.1.2 for neonatal data; v.1.2.2 for pediatric data; Oguz et al., 2014), we designed an age-specific DWI and DTI Quality Control protocol. The DTIPrep neonate-specific and pediatric-specific protocol parameters included criteria for correct DWI volume dimensions and gradient direction orientation, threshold values for detecting slice-wise intensity change, and excessive motion. After completion of the automatic QC processing to correct for motion and eddy currents, we visually inspected gradient volumes to manually identify those with remaining artifacts (see Figure 2). Gradient volumes affected by artifacts were excluded. As the diffusion acquisition employed a twice refocused sequence, rather than a polarity encoding sequence, no susceptibility correction was performed.

Following the above QC procedure, we computed diffusion tensors using weighted least squares fitting *Via* the DTIProcess software suite (Goodlett et al., 2009). Incorrect tensors with negative eigenvalues were corrected by replacing them with the nearest valid tensor. We calculated the tensor eigenvalues to obtain the diffusion properties, including Fractional Anisotropy (FA), Mean Diffusivity (MD), Axial Diffusivity (AD), and Radial Diffusivity (RD).

### Brain Masks

**FIGURE 3:**
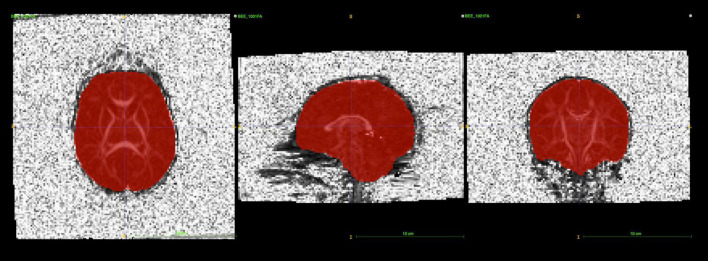
itk-SNAP allows users to visualize brain masks as a binary label image overlaying the b0 or FA image to perform manual editing. The main purpose of this step is to remove parts of the brain mask that cover any noisy, high-intensity voxels near the skull.

Brain masks for neonates were created from the baseline b0 image. An initial mask was created *Via* the Brain Extraction Tool (BET2; v.4.1.4) with the average b0 image as the input. Manual editing of these initial masks using itk-SNAP (Yushkevich et al., 2006) proved to be necessary (see Figure 3), in particular, areas of the frontal lobe were consistently underestimated in neonates.

Brain masks for the older participants were performed using the isotropic DWI image (the geometric average over all diffusion weighted images at b = 1000), instead of the average b0 image, as input to BET2. This choice provided more accurate initial masks than those generated from the baseline images for this age group. This also significantly reduced the amount of time required for manually editing the 1 and 2-year-old data.

For both the neonate and the pediatric data, the creation of brain-masked tensor images by applying the brain masks during the tensor estimation process served as the final pre-processing stage prior to atlas building.

### Atlas Building

Atlas building was performed *Via* DTIAtlasBuilder, which is an in-house developed, publicly available tool. In the first step of the atlas building, a prior FA template is employed as an initial reference frame for affine alignment. We employed a rescaled (1 mm × 1 mm × 1 mm resolution, size rescaled to neonate/pediatric setting) version of the JHU Eve atlas FA image (Geng et al., 2012b). Following affine alignment of the histogram normalized FA images, a first affine DTI atlas was computed by averaging this initial affinely aligned DTI data. All datasets were then again affinely registered to this initial affine DTI atlas. In its second step, DTIAtlasBuilder creates an unbiased diffeomorphic FA atlas *Via* joint, group-wise diffeomorphic atlas building. The resulting deformation fields are then applied to the tensor images using the preserving the principal direction (PPD) algorithm (Alexander et al., 2001), which were then averaged to yield the initial deformable DTI atlas. Finally, all DTI images are then individually re-registered *Via* symmetric diffeomorphic registration with ANTS registration tool (Verde et al., 2014). As above, the resulting displacement files are applied to the tensor images and the final deformable DTI atlas is computed by averaging all the warped tensor images.

During the construction of the DTI atlases, critical quality control of registration accuracy across all participants occurred at all three steps of the pipeline. Specifically, to ensure an appropriate atlas computation, we excluded any participant’s data that imperfectly aligned with the atlas in either the affine, group-wise warping, or individual warping steps (2 for neonate atlas, and 2 for pediatric atlas, all at groupwise warping and subsequent individual warping). It is noteworthy that a larger number of datasets were visually flagged during this quality control steps and then corrected (brain masking, affine registration initialization) prior to rerunning the atlas building procedure. This level of quality control ensured optimal SNR and contrast in the final DTI atlas.

### Tractography

White matter fiber tractography was applied to the DTI atlases built in the prior step. We employed 3D Slicer’s “Tractography Label Map Seeding” Module (Fedorov et al., 2012; Verde et al., 2014; neonates: version 4.3.0; pediatric: version 4.3.2) for this purpose. White matter tracts were seeded either using atlas-based, predefined ROIs or by manual tracing-based ROIs. All fiber bundles were reconstructed bilaterally except for the Corpus Callosum (CC), which is in interhemispheric tract. Four fiber bundles were divided into separate tracts based on tracking to predefined cortical surface targets (Oishi et al., 2010). A total of 47 tract segments were obtained for the neonatal DTI atlas (see Lee et al., 2015). The protocol used to generate each bundle and identify each segment is detailed in the supplement of Lee et al. (2015). For the pediatric atlas, the neonate atlas tracts served as the predefined ROIs after atlas co-registration.

For fiber bundles reconstructed in the neonatal atlas, tractography stopped where the FA value for the single tensor was less than 0.08 or the radius of curvature fell below 0.5 mm. In contrast, for the pediatric atlas, tractography stopped where the FA value was less than 0.1.

**FIGURE 4:**
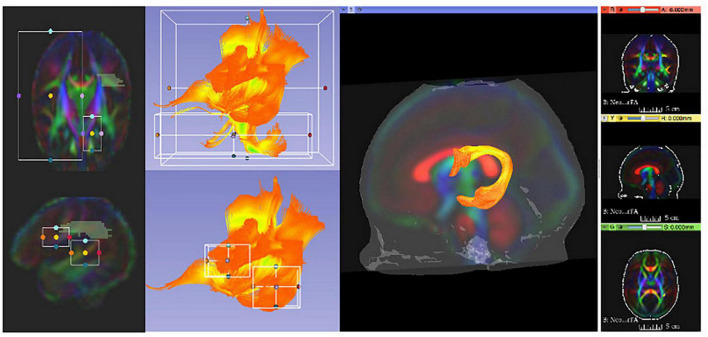
3D Slicer enables users to clean initial fiber bundles *Via* tract selection and editing modules. Additionally, it provides an interactive 3D viewing experience, which aids users to quality control white matter tracts that are being manually edited.

It is noteworthy that this fiber tracking procedure was designed to be overly generous in order to generate a tracking result for each bundle that minimally includes that bundle, but also includes a significant number of fibers from other bundles. Thus, by design, this tracking generated initial fiber bundles that required thorough cleaning and editing to dissect anatomically accurate reconstructions of white matter pathways. This was accomplished using a multi-stage filtering procedure. Initial fiber bundles were pre-cleaned by tract selection and editing using 3D Slicer (see Figure 4). The pre-cleaned fiber bundles underwent cluster based filtering *Via* FiberViewerLight (FVL; Verde et al., 2014). We applied all available clustering methods (in sequence: Length, Gravity, Hausdorff, and Mean Clustering) to remove outliers in FVL.

### Cropping the Fornix Tract

**FIGURE 5:**
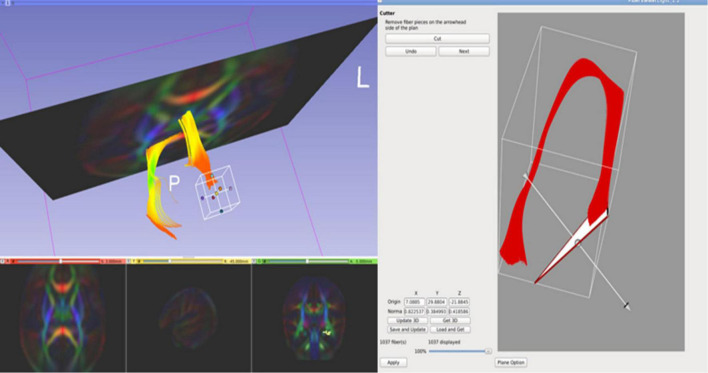
FiberViewerLight’s Cutter tool is useful when cropping certain regions of white matter tracts. We used this tool to crop the regions of the fornix that were determined to be artifactual streamlines.

In the case of the fornix, for both the neonatal and pediatric DTI atlas, the dorsal component incorrectly extended beyond the hypothalamus as far as the optic nerve for both left and right hemispheric tracts. This tracking artifact could not be removed simply by fiber filtering, but rather we manually shortened the fibers to exclude parts of the tracts. Using FVL, we cropped the regions of the fornix that were determined to be artifactual (see Figure 5). As a result, the finalized fornix tract segments follow the anatomically appropriate trajectory, with dorsal streamlines terminating in the hypothalamus and the ventral trajectory reaching the temporal pole in its course through the length of the hippocampus.

### Arcuate and Superior Longitudinal Fasciculus Tracts With Tract-Specific Atlas

For the arcuate and the superior longitudinal fasciculus (SLF) tractography, it was necessary to compute an arcuate-specific DTI atlas due the large inter-subject variance in arcuate morphology with a significant number of subjects missing larger sections of the fronto-temporal arcuate fiber (Catani and Mesulam, 2008). For this, we averaged the warped tensor images of only those participants where we could interactively identify arcuate tracts in each individual tensor image. This arcuate-specific atlas is thus a partial atlas in the same space as the full atlases, but with better contrast in the regions of the arcuate and SLF. The arcuate and SLF tractography were then performed on the arcuate-specific atlas.

## Results

### Diffusion Tensor Imaging Atlas and Tractography

**FIGURE 6:**
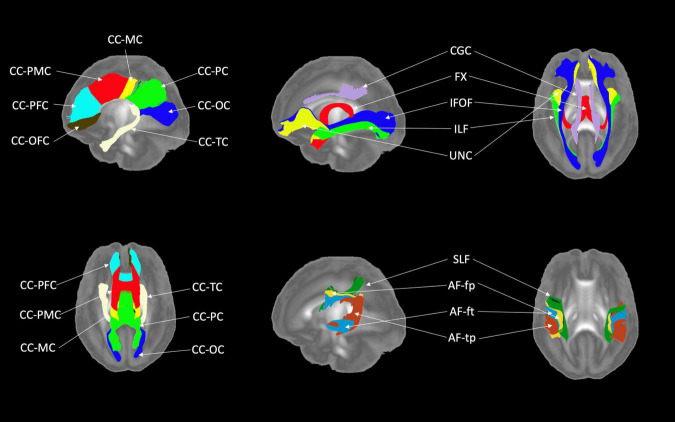
White matter tracts of the neonatal atlas. (Left top and bottom) Corpus callosum (CC); orbital frontal cortex (OFC); prefrontal cortex (PFC); premotor cortex (PMC); motor cortex (MC); parietal cortex (PC); occipital cortex (OC); tapetum (TC). (Top right) Blue = Inferior frontooccipital fasciculus (IFOF), purple = Cingulum superior part (CGC), yellow = Uncinate fasciculus (UNC), red = Fornix (Fx), green = Inferior longitudinal fascicles (ILF). (Bottom right) Green = Superior longitudinal fasciculus II (SLF), yellow = Arcuate fasciculus frontoparietal (AF-fp), red = Arcuate fasciculus temporoparietal (AF-tp), blue = Arcuate fasciculus frontotemporal (AF-ft). The underlying visualization image is a 3D volume rendering of the atlas FA map.

**FIGURE 7:**
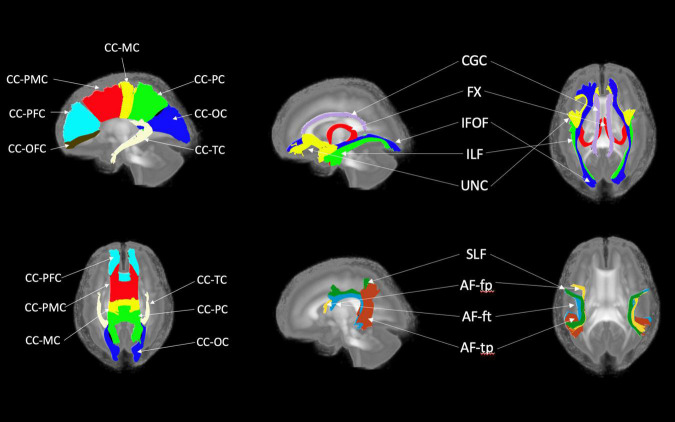
White matter tracts of the pediatric atlas. (Left top and bottom) Corpus callosum (CC); orbital frontal cortex (OFC); prefrontal cortex (PFC); premotor cortex (PMC); motor cortex (MC); parietal cortex (PC); occipital cortex (OC); tapetum (TC). (Top right) Blue = Inferior frontooccipital fasciculus (IFOF), purple = Cingulum superior part (CGC), yellow = Uncinate fasciculus (UNC), red = Fornix (Fx), green = Inferior longitudinal fascicles (ILF). (Bottom right) Green = Superior longitudinal fasciculus II (SLF), yellow = Arcuate fasciculus frontoparietal (AF-fp), red = Arcuate fasciculus temporoparietal (AF-tp), blue = Arcuate fasciculus frontotemporal (AF-ft). The underlying visualization image is a 3D volume rendering of the atlas FA map.

**FIGURE 8:**
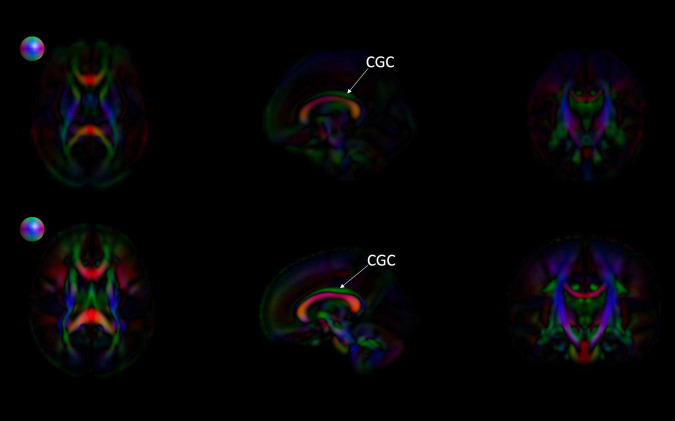
Comparing the EBDS neonatal and pediatric atlases. The images are the color-encoded orientation FA maps in axial, sagittal, and coronal slices. The top images are the neonatal atlas, and the bottom images are the pediatric atlas. From the sagittal view, we can observe that the cingulum (CGC) has higher FA due to the greater degree of myelination in the pediatric atlas. Furthermore, given the significantly lower SNR in the neonate DTI, its atlas appears less sharp than the pediatric DTI atlas. Spheres next to the axial view of atlases represent color coding for directional information.

**FIGURE 9:**
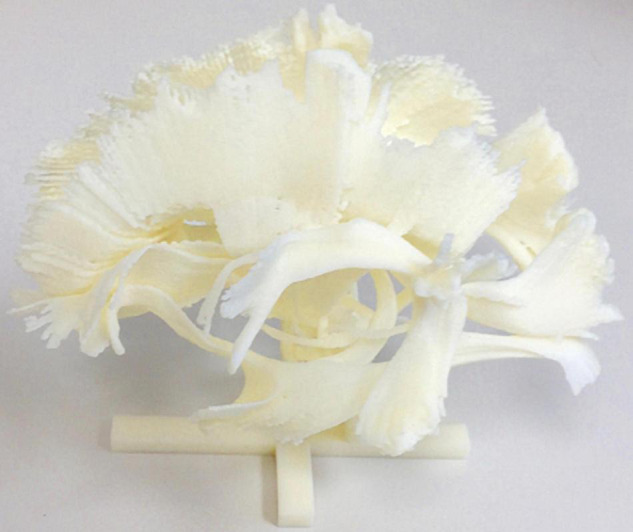
A 3D model of white matter tracts that were generated from the EBDS pediatric atlas for educational use.

Figures 6, 7 show 3D visualizations of the major white matter tracts in the neonatal and pediatric DTI atlas space. We were able to generate major white matter tracts from both the neonatal and pediatric DTI atlases including the uncinate fasciculus (UNC), inferior fronto-occipital fasciculus (IFOF), corpus callosum (CC), fornix (Fx), and inferior longitudinal fasciculus (ILF). Constructing a tract-specific atlas enabled us to reconstruct AF and SLF tracts in both atlases. In addition, we generated tract segments from CC based on trajectories to predefined cortical surface targets that correspond to putative functionally related regions. We observed that several tracts, including the cingulum (CGC) as illustrated in Figure 8, extended further toward the frontal cortex in the pediatric atlas compared to the CGC from the neonatal atlas. This is due to higher degree of myelination in the pediatric atlas and the consequently higher FA values. Given the lower SNR of the neonatal DTI, the individual dataset appears less sharp in the neonate, this leads to lower registration accuracy in neonates, as compared to older subjects and hence the atlas is not as sharp as older subjects. For educational use, we also generated a 3D printable model of the fiber tracts of the pediatric atlas that includes a stand (see Figure 9). These atlases alongside the white matter fiber tracts and 3D printable model are available publicly. See resource links at the end of this article and supplemental materials in Lee et al. (2015).

This tractography necessitates considerable interactive, manual efforts, both for the tractography ROIs as well as for the fiber cleaning/editing. While no exact book keeping was performed, we estimate that the full procedure took more than 12 months for the comprehensive set of fiber tracts, leading to several versions for each fiber bundle until a final, acceptable version was achieved. This significant effort highlights the need for the public dissemination of our DTI fiber atlases to facilitate efficient infant DTI studies. For the list of white matter structures, software terminology, and abbreviations, see link in supplementary materials for Appendix from Lee et al. (2015).

Our infant DTI atlases are intended to be used in two ways as described below. In our own studies we favor the second/study-specific option (see also section “Discussion”) as a study-specific DTI atlas provides the optimal analysis reference space, though at significant effort cost:

1. As a reference analysis space for infant studies, where subject DTI data is mapped directly into this atlas space, for example *Via* our DTI-Reg Slicer module, and diffusion properties are extracted along the atlas fiber tracts for statistical analysis.
2. As a fiber reference space that is employed to determine fiber tracts in a study-specific DTI atlas *Via* an automated tractography approach, such as our AutoTract tool (Prieto et al., 2016; Lam et al., 2018).

## Discussion

Numerous studies have used the UNC Utah NAMIC DTI framework alongside our DTI atlases to examine brain development in neonates and infants. With few changes to the overall framework, these studies followed the methods employed in this article. In Lee et al. (2015), a fiber tract based study on the heritability of white matter structural development was conducted by mapping all study datasets into the pediatric atlas. Rasmussen et al. (2017) conducted a study to examine normative white matter maturation in humans and introduced a new Maturation Index (MI). Our neonate DTI atlas was applied to build a study-specific DTI atlas in the same reference space and to initialize tractography in that study-specific atlas. Swanson et al. (2017) demonstrated the use of the pediatric DTI atlas to find associations between language and white matter fiber bundles (i.e., splenium, arcuate, uncinate, and left inferior longitudinal fasciculi) using similar processing. Collectively, these studies and several others (see Girault et al., 2019; Short et al., 2019; Sket et al., 2019; Stephens et al., 2020) demonstrate the use of our DTI atlases, in combination with the UNC Utah NAMIC DTI framework, to quantitatively analyze normative patterns of early white matter brain development in a consistent reference space.

While this framework was used to examine normative brain development in the above mentioned studies, several studies have also used the DTI atlas as a reference to identify early indicators of atypical brain development. In a study conducted by Gupta et al. (2015), the authors investigated the use of neonate DTI data to quantify the disease progression in infants with the early form of Krabbe disease. In this study, the individual image data acquired from Krabbe cases were registered into the neonate DTI atlas, and they assessed the DTI parameters to compare and predict the longitudinal development in four functional domains. Our neonatal and pediatric atlases were also used as a reference space in a longitudinal study examining the association between white matter development and restricted and repetitive behaviors in autism (Wolff et al., 2017). Researchers in another study examined white matter atypicality associated with Fragile X Syndrome (FXS; Swanson et al., 2018). Similarly, Ahn et al. (2019) investigated the time frame in which neural abnormalities associated with schizophrenia first emerge using our pediatric atlas. Rasmussen et al. (2019) also applied a similar processing pipeline to study the relationship between the maternal concentrations of proinflammatory cytokine interleukin-6 (IL-6) and alterations in the structural connectivity of frontolimbic circuitry. Jha et al. (2016) examined the effects of prenatal exposure to selective serotonin reuptake inhibitors on neonatal white matter microstructure. Together, these studies highlight the utility of age-appropriate pediatric DTI atlases to enable early detection of neurodegenerative diseases or identification of atypicality in the earliest stages of brain development.

A limitation of our DTI atlases is that they are based on the registration of intensity-normalized FA images and thus the registration did not incorporate the local fiber orientation in the atlas generation process. Future atlases could improve upon our method by using a tensor-based registration algorithm which is superior to using scalar images for cross-subject image alignment. This can be done for example *Via* the DTI-ToolKit (DTI-TK) tools in the DTI-reg processing procedure which allows for registration based on tensors.

Furthermore, as some of the studies introduced in the previous section have shown, future research could utilize our neonatal and pediatric atlases as well as the library of white matter tracts specific to these atlases for the study of both typical development and investigations of clinical pathology. Providing resources for clinical implementation is essential as it enhances the diagnostic utility of neuroimaging and quantitatively identifies aberrant developmental patterns (Oishi et al., 2013). Thus, our atlases can serve as a valuable resource in clinical fields by alleviating the limited availability of appropriate age-specific neuroimaging data.

In summary, this paper presents the generation of a new public resource for neuroimaging studies of early brain development. We have constructed two DTI atlases, one for neonates and another for pediatric populations. These atlases consist of both reference tensor templates, as well as a library of major white matter tracts delineated on these atlases. We encourage the field to use these high-quality standardized DTI brain templates and the associated white matter fiber bundles for studies of both typical and atypical brain development. Understanding of early brain maturation is still in its infancy and much remains to be discovered about normative brain development. The utility of these atlases also extends to clinical scenarios, where the use of such templates can enhance the diagnostic utility of neuroimaging and quantitatively identify aberrant developmental patterns. Furthermore, the white matter tract library and the 3D printed visualization of these tracts can also serve as interactive educational tools in multidisciplinary settings.

## Online Resources

The EBDS pediatric DTI atlases are available at the following link: https://www.nitrc.org/projects/uncebds_neodti NAMIC DTI Analysis Framework available at the following link: https://www.nitrc.org/projects/namicdtifiber/DTIAtlasBuilder: https://www.nitrc.org/projects/dtiatlasbuilder.

## Data Availability Statement

The datasets presented in this article are not readily available because NIH holds the rights to these data. Requests to access the datasets should be directed to JHG, john_gilmore@med.unc.edu.

## Ethics Statement

The studies involving human participants were reviewed and approved by Institutional Review Board at the University of North Carolina at Chapel Hill. Written informed consent to participate in this study was provided by the participants’ legal guardian/next of kin.

## Author Contributions

SJS, DJ, RJS, and MS wrote the manuscript. RLS, JBG, and JHG provided insightful comments and reviewed content. All authors contributed to the article and approved the submitted version.

## Author Disclaimer

The views expressed in this manuscript are those of the authors and do not necessarily represent the opinions or position of listed funding agencies.

## Conflict of Interest

The authors declare that the research was conducted in the absence of any commercial or financial relationships that could be construed as a potential conflict of interest.

## Publisher’s Note

All claims expressed in this article are solely those of the authors and do not necessarily represent those of their affiliated organizations, or those of the publisher, the editors and the reviewers. Any product that may be evaluated in this article, or claim that may be made by its manufacturer, is not guaranteed or endorsed by the publisher.
